# Quantum magnetism in strongly interacting one-dimensional spinor Bose systems

**DOI:** 10.1038/srep10675

**Published:** 2015-06-15

**Authors:** Amin Dehkharghani, Artem Volosniev, Jonathan Lindgren, Jimmy Rotureau, Christian Forssén, Dmitri Fedorov, Aksel Jensen, Nikolaj Zinner

**Affiliations:** 1 Department of Physics and Astronomy, Aarhus University, DK-8000 Aarhus C, Denmark; 2 Department of Fundamental Physics, Chalmers University of Technology, SE-412 96 Göteborg, Sweden; 3 Department of Physics and Astronomy, University of Tennessee, Knoxville, TN 37996, USA; 4 Physics Division, Oak Ridge National Laboratory, Oak Ridge, TN 37831, USA

## Abstract

Strongly interacting one-dimensional quantum systems often behave in a manner that is distinctly different from their higher-dimensional counterparts. When a particle attempts to move in a one-dimensional environment it will unavoidably have to interact and ‘push’ other particles in order to execute a pattern of motion, irrespective of whether the particles are fermions or bosons. A present frontier in both theory and experiment are mixed systems of different species and/or particles with multiple internal degrees of freedom. Here we consider trapped two-component bosons with short-range inter-species interactions much larger than their intra-species interactions and show that they have novel energetic and magnetic properties. In the strongly interacting regime, these systems have energies that are fractions of the basic harmonic oscillator trap quantum and have spatially separated ground states with manifestly ferromagnetic wave functions. Furthermore, we predict excited states that have perfect antiferromagnetic ordering. This holds for both balanced and imbalanced systems, and we show that it is a generic feature as one crosses from few- to many-body systems.

The interest in one-dimensional (1D) quantum systems with several interacting particles arguably began back in 1931 when Bethe solved the famous Heisenberg model of ferromagnetism^1^, but it was only in the 1960 s that people realized that the techniques invented by Bethe could be used to solve a host of different many-body models^2^,^3^,^4^,^5^,^6^. It was subsequently realized that many 1D systems have universal low-energy behaviour and can be described by the paradigmatic Tomonaga-Luttinger-Liquid (TLL) theory^7^,^8^,^9^. This opened up the field of one-dimensional physics, which has remained a large subfield of condensed-matter physics ever since^9^,^10^. Recently, there has been a great revival of interest in 1D systems due to the realization of 1D quantum gases in highly controllable environments using cold atomic gases^11^,^12^,^13^,^14^,^15^,^16^,^17^. This development implies that one may now experimentally realize 1D systems with bosons or fermions and explore the intricate nature of their quantum behaviour.

A recent frontier is the realization of multi-component systems^18^ in order to study fundamental 1D effects such as spin-charge separation^19^. While this effect is usually associated with spin 1/2 fermions, it turns out that it can also be explored in Bose mixtures (two-component bosonic systems) where the phenomenon can be even richer as there can be interactions between the two components (inter-species) and also within each component separately (intra-species)^10^,^20^,^21^. The latter is strongly suppressed for fermions due to the Pauli principle. In the case where the intra- and inter-species interactions are identical it has been shown that a ferromagnetic ground state occurs^22^,^23^. Generalizing to the case of unequal intra- and inter-species interactions may be possible, but since the proofs and techniques rely on spin algebra and representation theory, they cannot be used to obtain the full spatial structure of general systems and other approaches are therefore needed. Here we consider the limit where the inter-species dominates the intra-species interactions. This regime has been explored in recent years for small systems using various few-body techniques^24^,^25^,^26^,^27^,^28^,^29^ and behaviour different from strongly interacting fermions or single-component bosons can be found already for three particles^29^. From the many-body side, the system is known to have spin excitations with quadratic dispersion,^30^,^31^,^32^,^33^ which can be shown to be a generic feature of the ‘magnon’ excitations above a ferromagnetic ground state^34^,^35^. This goes beyond the TLL theory and it has been conjectured that a new universality class (‘ferromagnetic liquid’) emerges in this regime^36^,^37^,^38^,^39^,^40^.

Here we provide a particularly clean realization of a ‘ferromagnetic’ system confined in a harmonic trap. Using numerical and newly developed analytical techniques we obtain and analyze the exact wave function. This allows us to explore the crossover between few- and many-body behaviour, and to demonstrate that the strongly interacting regime realizes a perfect ferromagnet in the ground state, while particular excited states will produce perfect antiferromagnetic order. In the extremely imbalanced system, with one strongly interacting ‘impurity’, we find both numerically and analytically that the impurity will always move to the edge of the system. This is in sharp contrast to fermionic systems where the impurity is mainly located at the center^41^. Our work provides a rare and explicit example of perfect ferro- or antiferromagnetism using the most fundamental knowledge of a quantum system as given by the full wave function.

## Results

### Energetics and wave functions

Our two-component bosonic system has 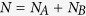 particles split between 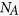 and 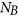 identical bosons of two different kinds. All 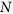 particles have mass 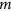 and move in the same external harmonic trapping potential with single-particle Hamiltonian 
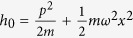
, where 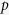 and 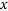 denote the momentum and position of either an 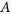 or 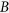 particle and 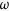 is the common trap frequency. The trap provides a natural set of units for length, 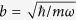, and energy, 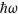, which we will use throughout (here 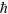 is Planck’s constant divided by 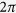). We assume short-range interactions between 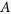 and 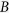 particles that we model by a Dirac delta-function parameterized by an interaction strength, 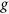, i.e.
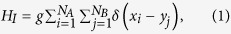
where 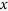 and 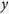 denote the coordinates of 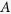 and 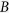 particles, respectively. The intraspecies interaction strengths are assumed to be much smaller than 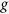 and we will therefore neglect such terms. To access the quantum mechanical properties of our system we must solve the 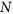-body Schrödinger equation. This will be done using novel analytical tools and using exact diagonalization. In the latter case we have adapted an effective interaction approach that has recently been succesfully applied to fermions in harmonic traps^42^,^41^ (see the Methods section for further details). The analytical and numerical methods allow us to address up to ten particles, which is larger than most previous studies not based on stochastic or Monte Carlo techniques.

**Figure 1:**
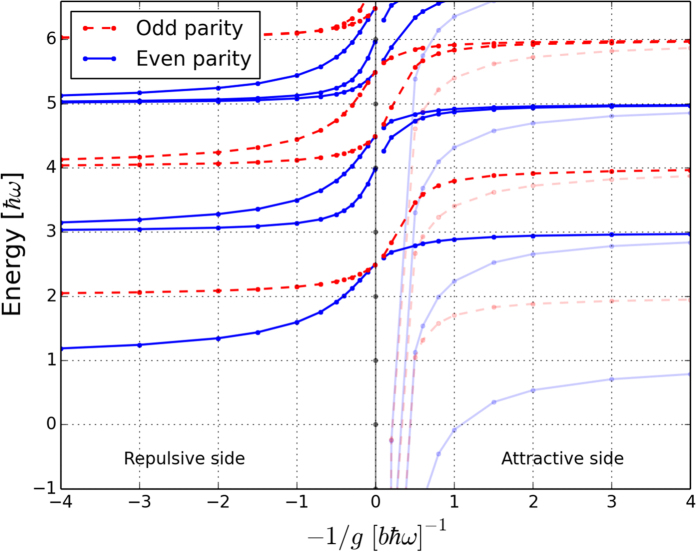
Three-body spectral flow. The energy spectrum of two 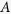 and one 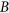 particle as a function of interaction strength, 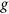, obtained by numerical calculations. In the limit 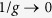, the ground state becomes doubly degenerate and has half-integer energy. The contribution from center-of-mass motion has been removed. For visibility, we have dimmed states from the attractive side that diverge to large negative energies close to 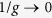.

The simplest non-trivial case is the three-body system which has two 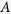 and one 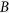 particle. The energy spectrum is shown in Fig. 1 as a function of 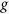. The most interesting feature to notice is the ground state behaviour as 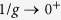. Here, an odd and an even state become degenerate at an energy of 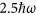. This should be contrasted to the behaviour of single-component bosons or two-component fermions which will always have energies that are an integer times 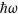 when 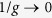. Furthermore, we notice how the two states that merge at 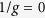 become two excited state branches on the attractive side of the resonance but the even parity state remains the lower one. This is opposite to the behaviour of fermions^43^ where the hierarchy of states is inverted at 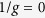. The ground state for large and negative 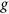 is very different as it contains deeply bound molecules, which we will not consider further. The fractional energies in the spectrum can be explained by a schematic three-body model and in stochastic variational calculations^29^. This provides a hint that larger systems could also display fractional energy states in the strongly interacting limit and begs the question as to what spatial configurations such states correspond to.

We will now show that the fractional energy states are generic for strongly interacting two-component bosons in 1D and, importantly, for the ground state they realize perfect ferromagnetic behaviour irrespective of whether the system is balanced (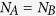) or not. The term perfect ferromagnetic behaviour implies that we have a full spatial separation of the two components in the exact ground state wave function of the system, i.e. the probability to find only 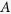 on one side and only 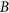 on the other side of the system is not just dominant, it is exactly *unity*. The ground state has only a single ‘domain wall’ at which an 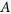 and a 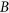 particle are neighbours. As a consequence, imagine that you detect an 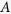 particle on the left (right) side of the system, then you can immediately conclude that all the 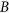 particles reside to the right (left) of this 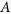 particle.

### Balanced systems

**Figure 2:**
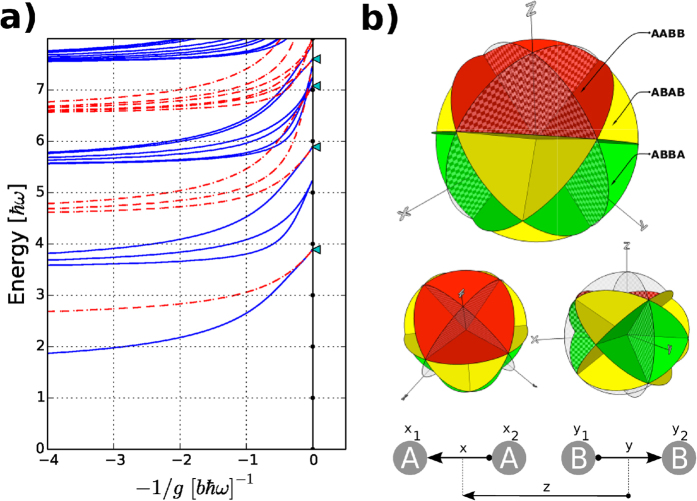
**Balanced four-body system. a**) Energy spectrum for 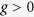 for two 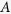 and two 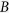 particles. The 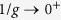 limits are analytically known and indicated by triangles. Even parity states are in blue solid while odd parity states are in red (dot-dashed). **b**) Three-dimensional representation of the coordinate space on which the four-body wave function is defined when the center-of-mass position is removed. The specific Jacobi coordinates used are shown at the bottom. The solid coloured circular planes indicate the planes where an 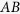 pair overlap. The wave function must vanish on these planes in the limit where 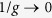. The checkerboard coloured circular planes are reflection planes for a pair of identical particles (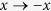 and 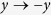). The red region has twice the volume of the green region and four times that of the yellow region. The smaller figures in the middle show the same regions viewed from different angles for clarity.

We first consider a four-body system that has two 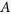 and two 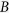 particles. The energy spectrum for 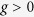 is shown in Fig. 2a). A striking feature is the two-fold degenerate ground state for 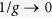 that has a non-integer energy similar to the three-body problem. In this strongly repulsive limit, the system realizes a perfect spatially ferromagnetic quantum state as we will now demonstrate analytically.

First we note that the center-of-mass motion of the four-body system can be separated and thus ignored. This leaves three Jacobi coordinates to describe the system. The details of these reductions can be found in the Methods section below. In Fig. 2b) we show the space of the Jacobi coordinates and highlight all the planes at which an 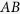 (solid planes), an 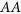 or a 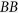 (checkerboard planes) pair of particles overlap. The main observation is that as 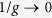, the wave function must vanish on all the solid planes in Fig. 2b) and we arrive at the disconnected regions shown with different colours. The particles become effectively impenetrable and we may characterize the wave function by specifying the amplitudes of all possible spatial configurations of the four particles. These regions correspond to specific orderings on a line of the four particles. In particular, the large (red) region dominating the figure corresponds to spatial configurations 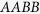 or 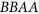. The green region occupies half the spatial volume of the red and corresponds to 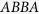 or 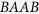, while the yellow region has one-fourth the volume of the red region and corresponds to 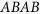 or 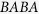 configurations. A wave function that vanishes on all 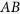 interfaces may now be constructed in each of these regions. However, it is immediately clear that it will have lower energy when it can spread over a larger volume. We thus conclude that the doubly degenerate ground state at 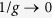 has the structure 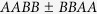 (taking into account the parity invariance of the Hamiltonian).

As discussed in the Methods section, one may solve a simple wave equation in the red region and obtain the ground state energy to arbitrary precision. The triangles at the 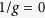 line in Fig. 2a) show the energies obtained in this manner. We reproduce both the ground state and a set of excited states. All of these have fractional energies and all of them are perfectly ferromagnetically ordered. The remaning states of the spectrum can be obtained by solving in the other regions of Fig. 2b). Note that states with amplitude exclusively in the yellow regions are perfectly spatial antiferromagnetic, 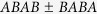, and have energies (n + 1/2) 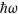 with integer 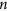. They are the only parts of the spectrum which can be constructed by starting from identical fermions using Girardeau’s mapping techniques^44^. The arguments presented here are neither restricted to 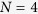 nor to a harmonic trapping potential and hold for any 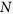 and any shape of the external confinement. They hinge only on the fact that the 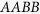 or 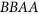 configurations occupy the largest volume. For instance, for 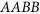 the four regions 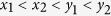, 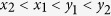, 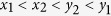 and 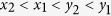 are adjacent regions and are connected by Bose symmetry of each component, thus they make up the connected upper red region (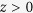) of the coordinate space in Fig. 2b). For 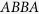 one finds that only 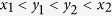 and 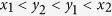 are adjacent and connected. This implies that the volume for 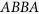 is half as large.

**Figure 3:**
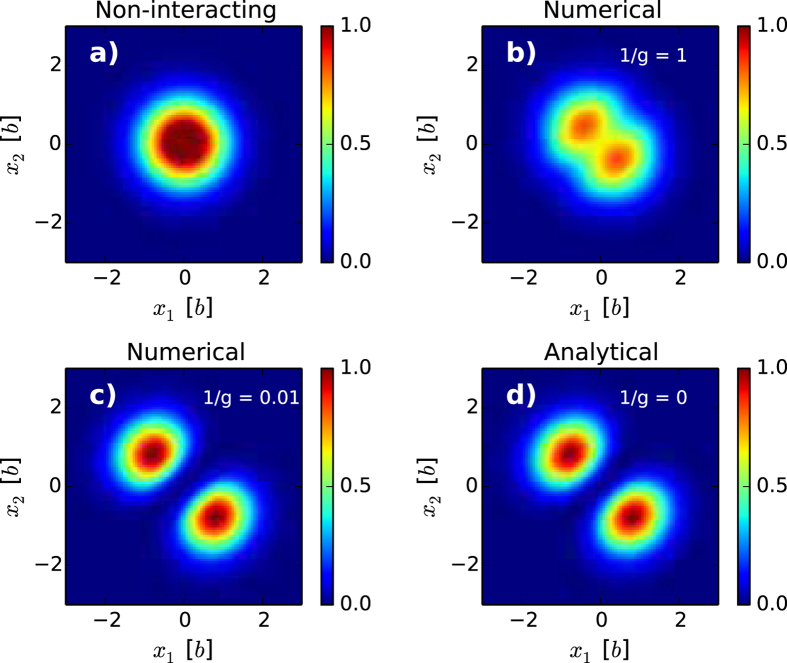
**Pair-correlation function for ground state of the balanced four-body system. a)** The non-interacting case where 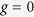. **b)** and **c)** Numerically calculated pair-correlation function at 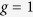 and 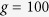. The phase separation starts slowly at 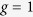 and the magnetic ordering is fully present at 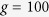. **d)** Analytically calculated pair-correlation function for 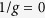. Notice how **c)** and **d)** are virtually indistinguishable.

In order to further study the correlation between the 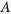 and 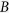 particle subsystems, one can use the pair-correlation function. The pair-correlation function measures the probability of finding a particle from one subsystem (say of type 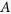) at position 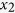 given that we know the position, 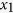, of a particle in the other subsystem (say of type 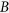). The pair-correlation function for the balanced 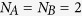 case is shown in Fig. 3a) for the case where 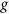 is zero and therefore no separation and hence no specific ordering is present, but as 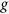 becomes strongly repulsive evidence of separation is seen as illustrated in Fig. 3b,c). In particular, Fig. 3c) shows that if we have a given particle from one subsystem situated on the negative 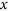-axis (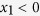) then a particle from the other subsystem is most likely to be found on the positive 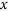-axis (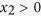) and vice versa. These numerical calculations are also supported by our analytical method for the four-body system at 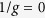. Fig. 3d) shows the analytical result that is virtually identical to the numerical results of Fig. 3c.

**Figure 4:**
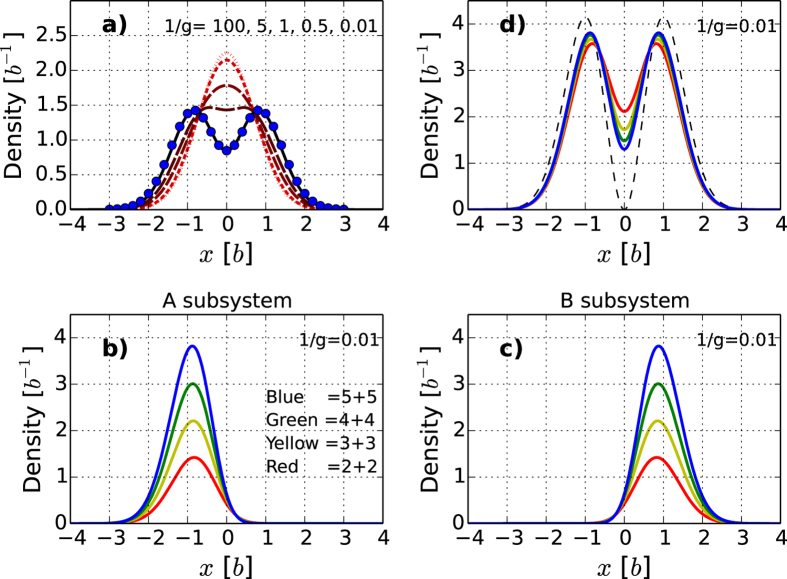
**Ground state densities of balanced systems. a**) Total density for 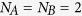 and different values of 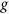. Dotted (red) line corresponds to 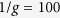 while the solid (black) line is for 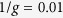. The dots show the analytical solution for 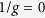. **b**) and **c**) Densities for an equal superposition (sum) of the (nearly) two-fold degenerate ground state at 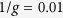 for 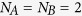, 3, 4, and 5. **b**) shows 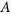 particles and **c**) shows 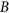 particles. **d**) Rescaled plot of the total density at 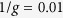. For 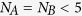 the density has been rescaled to a total density with 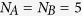. The dashed line corresponds to the density expected in the many-body limit 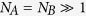.

Larger systems may in principle be handled in similar fashion by solving wave equations with proper boundary conditions and obtaining the fractional energies in the limit 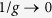. However, the increase in dimension of the problem makes this very difficult in practice. In order to further demonstrate that balanced systems have perfect ferromagnetic ground states irrespective of particle number, we have numerically computed the ground state densities for systems with 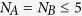 as shown in Fig. 4. Evidence of the separation of 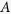 and 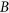 can be seen in the total density already in Fig. 4a) for 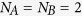 as 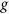 increases (note the perfect agreement with the analytical result in the limit 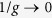). We expect the two degenerate ground states to have structure 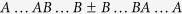. In order to prove this perfect ferromagnetic behaviour, we consider the odd and even superposition of the two degenerate states which we expect will yield states with exclusively 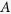 or 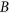 particles on either side of the system (corresponding to 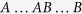 or 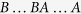). The corresponding densities are shown in Fig. 4b) and Fig. 4c) and beautifully confirm our expectations.

As the ground state for 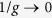 is spatially separated, one may speculate that it can be understood physically as two ideal Bose gases or ‘condensates’ sitting on either side of the system even in this strongly interacting limit. In Fig. 4d) we plot the densities in a rescaled fashion where we multiply by 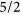, 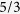, and 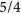 on the 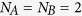, 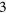 and 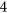 densities respectively. The convergence of the results toward the 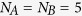 case indicates that the system does behave as two ideal Bose gases as the particle number grows. In the limit 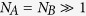, we would expect the overlap of the two gases to vanish as the energy cost of overlap goes to infinity. We therefore expect that the occupied mode in this large system limit is the first excited state of the harmonic trap which vanishes at the center. The dashed line in Fig. 4d) shows this state rescaled to 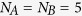. This analytical guess displays the same features as the numerical densities and we conclude that already for ten particles the many-body properties are emerging.

### Imbalanced systems

**Figure 5:**
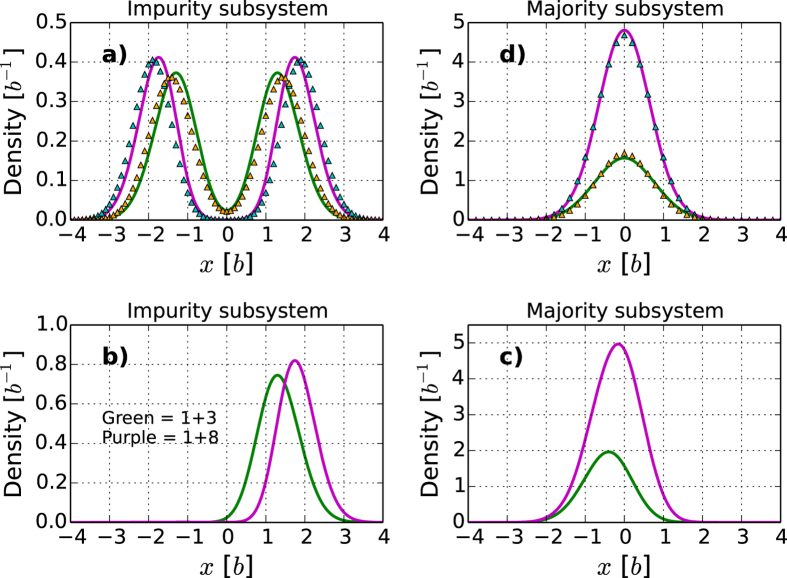
**Ground state densities of imbalanced systems. a**) Impurity density in an 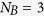 (green) or 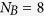 (purple) systems with 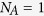. The analytical results for 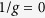 are shown as triangles. **b**) and **c**) as in Fig. 4b) and c) but for 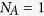 with 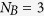 or 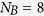. **d**) The density of the majority component (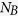). All numerical results have been obtained with 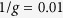.

The extremely imbalanced limit, where 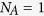 and 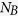 varies, provides a realization of a strongly interacting Bose polaron in 1D, i.e. an impurity that interacts strongly with an ideal Bose gas. In Fig. 5 we plot the densities of systems with 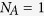 and 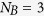 or 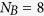. We see that the impurity sits at the edge of the system (Fig. 5a), while the majority component tends to occupy the center (Fig. 5d). We confirm the numerical results by employing an analytical model, which shows excellent agreement. The details can be found in the Methods section. To confirm that the wave function of the strongly interacting ground state has intrinsic phase separation, i.e. has the form 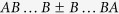, we plot the densities for a sum of the nearly degenerate ground states in Fig. 5b,c). As in the balanced case above, we find a perfectly separated ground state behaviour. For instance, if we locate the single 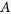 particle on one side of the trap, we would thus immediately know that all the 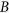 particles reside on the other side, and vice versa. This behaviour is opposite to the case where the 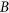 particles are identical fermions where the impurity resides mainly in the center of the system^41^. We have confirmed that this structure is also present for 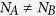 with 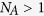, and it is therefore a generic feature that the two species are perfectly spatially separated (ferromagnetic) in the ground state for strong interactions.

A remarkable feature of the densities in Fig. 5b,c) is the movement of the centroids of the peaks with particle number. We clearly see the majority moving into the center and the impurity being pushed toward the edge. This demonstrates how an ideal condensate is being built in the center. For large 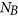 the energy per particle goes to 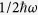, which implies a single-mode condensate that is becoming macroscopically occupied (see Methods section for details). The relative deviation between numerical and analytical energies is below three percent for 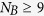. We thus have an analytic model for the crossover between the few- and many-body limit for the bosonic polaron in one dimension. This includes the external trap that is a reality of most experiments.

## Discussion

**Figure 6:**
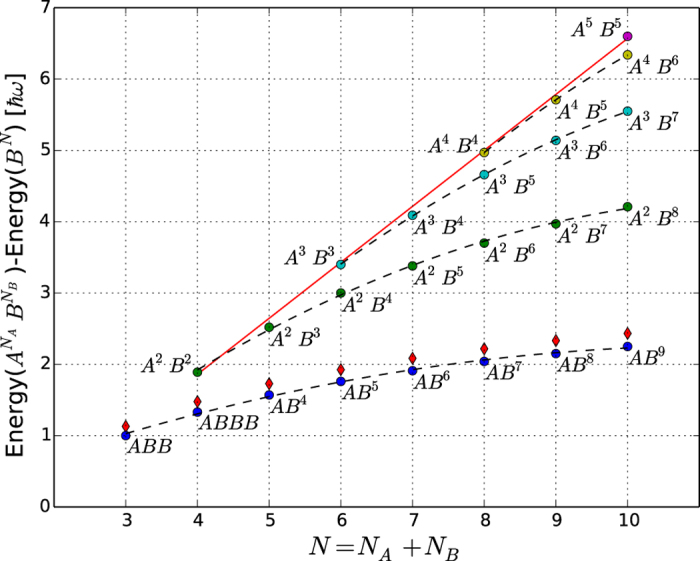
Ground state energies for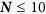. The filled circles show the ground state energy for 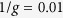 relative to the zero-point energy as given by a single-component system of the same size. Each point is marked with the number of particles using the notation 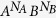. The diamonds are the results of the analytical method for the polaron case described in the Methods section. The dashed lines are quadratic interpolations for fixed number of 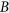 particles, while the solid line is an interpolation of the energy for the balanced systems.

We have shown that a mixture of two ideal Bose systems in one dimension has unusual properties when the inter-species interactions is strong. The systems have energies that are non-integer multiples of 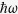. In Fig. 6 we show the ground state energies for 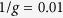 in systems with ten particles or less relative to a ground state with only 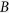 particles. Driving an 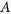 to 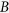 transition via radiofrequency spectroscopy would be a possible way to confirm the predicted energies in Fig. 6. This technique has been demonstrated for fermions in recent few-body experiments in 1D^17^. The results in Fig. 6 show that the energy per particle tends to saturate for large systems and that this happens faster the more imbalanced the system is. We also see that the balanced case has an almost linear energy dependence.

**Figure 7:**
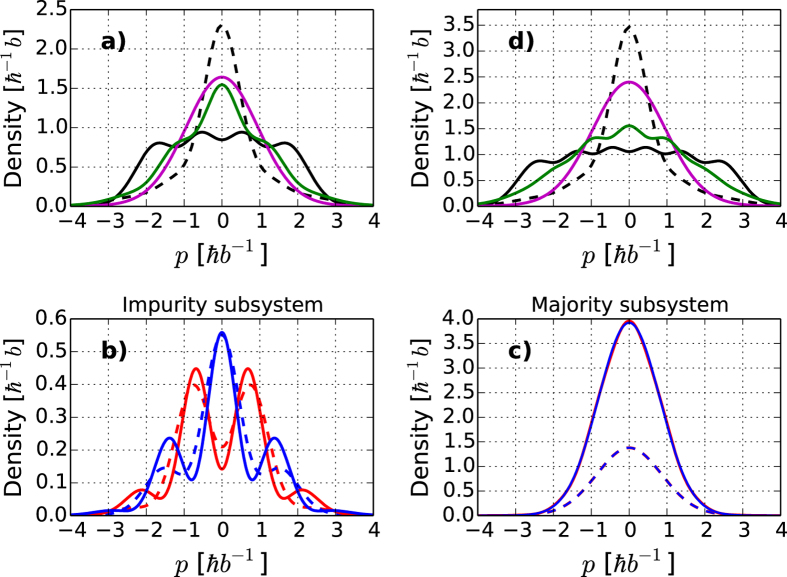
**Momentum distributions. a**) 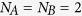 system. The even parity ground state is shown in purple, while the lowest excited state with antiferromagnetic structure is shown in green. For comparison the four-peak black curve represents identical fermions while the narrow peak dashed black curve shows hard-core bosons. **b**) Ground state impurity distributions for the even (blue) and odd (red) ground states with 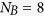 (solid) and 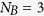 (dashed). **c**) Ground state majority distributions for 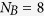 (solid) and 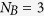 (dashed). The even and odd parity results coincide for the majority. **d**) The same as in **a**) for 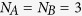. All curves in the plot have been obtained for 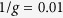.

The ferro- and antiferromagnetic states can also be detected by measuring momentum distributions. In Fig. 7a,d) we show the distributions for 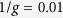 with 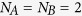 and 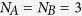, respectively. The purple solid line is the even parity ground state while the solid green line is the excited state with antiferromagnetic ordering. The striking difference of the two distributions implies that they should be easily identifiable in experiments. For comparison, the solid black line with a multi-peak structure shows the distribution for a system of identical fermions. Comparing the solid green and black curves in Fig. 7a,d) clearly demonstrates that, in spite of the fact that these two states have equal energy, the correlations are very different. In Fig. 7a) the dashed black line corresponds to the Tonks-Girardeau hard-core boson state^44^, which is also seen to be very different from the states discussed here. For imbalanced systems, we find that measuring the impurity momentum distribution, Fig. 7b), yields information about the parity of the state. On the other hand, the majority distributions in Fig. 7c) are identical in the two opposite parity ground states. A characteristic feature seen in Fig. 7b) is the development of oscillatory structure as the number of majority particles increases and pushes the impurity further out in the trap, see also Fig. 5b). A third technique for experimentally addressing the systems we study is controlled tunneling as the trap is gradually lowered^16^ (see Methods below).

The separation of components in the ground state for strong interactions is intrinsic to both balanced and imbalanced mixtures, as is the presence of other spatial configurations in specific excited states. Furthermore, the magnetic behaviour discussed above is not connected to the harmonic confinement and should be seen in an arbitrary confining geometry. While we have studied the balanced and the extremely imbalanced limits here, we have checked numerically that the spatial separation of components is an intrinsic feature of the system for systems with ten or less particles. We therefore infer that this will hold also for larger systems, regardless of the population ratio. A simple physical picture can be given in terms of domain walls, i.e. points at which the two components interface. The system tends to minimize the number of domain walls and this principle can be used to understand the ferromagnetic ground state and predict the ordering in energy of other configurations.

In the paradigmatic two-component (spin 1/2) Fermi system, the ground state is never purely ferro- or antiferromagnetic for strong interactions^43^, and Bose mixtures therefore provide a unique set of quantum ground states for exploring and exploiting magnetic behaviour. The description of these systems clearly goes beyond the famous Bose-Fermi mappings^44^,^45^] and we provide not only numerical but also new analytical tools to fill this gap. Importantly, we demonstrate that the crossover from few- to many-body physics can be studied already at the level of ten particles.

## Methods

### Numerical method

We solve numerically the many-body Schrödinger equation by exact diagonalization with the full Hamiltonian projected onto a finite basis constructed from harmonic oscillator single-particle states. Each many-body basis state is written as a product of symmetrized states of 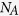 and 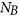 particles. The model space truncation is defined by an upper limit of the total energy.

Instead of the bare zero-range interaction in (1), we consider an effective two-body interaction in order to speed up the convergence of the eigenstates with respect to the size of the many-body basis. The effective potential is constructed in a truncated two-body space, and is designed such that its solutions correspond to exact two-body solutions given by the Busch formula^46^. As explained in detail in Refs. 41,42, this is achieved using a unitary transformation that involves the lowest eigensolutions given by the Busch formula. By construction, this unitary transformation approach will reproduce exact bare Hamiltonian results for the many-body system (both energy spectrum and wave functions) in the limit of infinite model space.

The excellent convergence property of this effective-interaction approach was demonstrated in Ref. 41 and is key to the quality of our numerical results and to our conclusions. In the construction of the effective interactions we benefit from having access to the exact two-body solutions for short-range interactions in harmonic traps. However, we stress that using numerical two-body solutions this approach can be generalized to study many-body systems in higher dimensions with finite-range interactions and in any trapping potential.

### Density and pair-correlation profiles

In the second quantization formalism the density profile is calculated by taking the expectation value of the number operator, 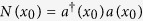 at position 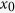, where 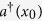 creates a particle at position 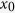 and 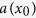 annihilates it. In other words, 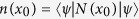, where 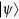 is the many-body basis state written as a product of symmetrized states of 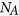 or 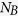 particles. In the same manner the pair-correlation profile is calculated between two particles from different subsystems. It is defined as the expectation value of two number operators, 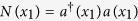 and 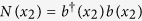, one from each subsystem, i.e. 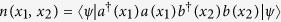.

In our analytical results we use the following expressions to obtain the density and pair-correlation from the 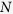-body wave function, 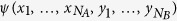. To get the density of 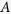 particles in the system we need to calculate
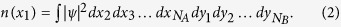


Likewise, to obtain the density of a 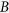 particle we integrate over all variables except 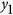 instead of 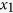. The 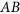 pair-correlation is obtained by calculating
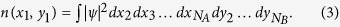


### Analytics for balanced systems

Here we outline the calculational procedures required to obtain the exact solutions for the 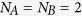 four-body system in the 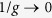 limit. The method can in principle be extended to larger systems, but it becomes increasingly difficult. In the next subsection we provide an alternative method that works well for larger systems in the imbalanced case.

Denote the 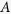 coordinates by 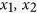 and the 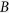 coordinates by 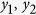, see Fig. 2b). The Hamiltonian is
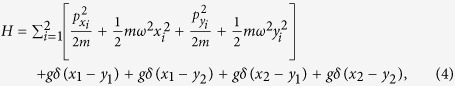
with 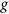 the 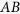 interacting coupling constant and we assume that the 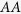 and 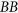 interactions vanish. We now perform an orthogonal coordinate transformation
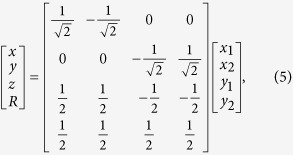
where 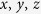 are as shown at the bottom of in Fig. 2b) while 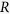 denotes the center-of-mass coordinate. The quadratic kinetic and harmonic oscillator terms in 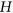 retain their form under this transformation and one may immediately separate the center-of-mass, 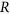, which can be ignored from now on. For the remaining three coordinates we switch to the usual spherical coordinate system, i.e. 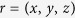, 
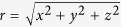
, 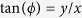, and 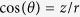. Carrying out these transformation we arrive at the relative motion Hamiltonian
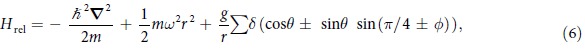
where the sum in the interaction term runs over the four combinations of signs in the argument of the delta function. The first two terms constitute a 3D harmonic oscillator with the well-known regular solution 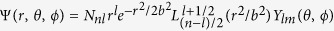, where 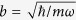 and 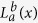 is the generalized Laguerre polynomial.

When 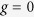, the angular functions, 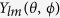, are the usual spherical harmonic functions with 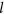 the total and 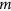 the projection angular momentum quantum number. However, in the limit 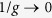 we have to enforce non-trivial boundary conditions whenever 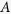 and 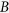 particles overlap. Let us focus on the region 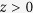 by restricting to 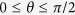 (solutions for 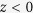 may be obtained by symmetry arguments or by considering instead 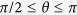). The arguments of the Dirac delta-functions in Eq. (6) vanish when
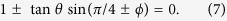


If we define 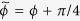, we have 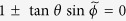 and 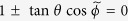. The regions defined by these conditions are illustrated in Fig. 2b). The solid red planes show exactly where the arguments of the interaction Dirac delta-functions have to vanish.

We now make the simple transformation 
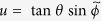
 and 
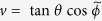
. In these new 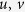 variables, the boundaries are simply 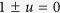 and 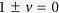, i.e. the function must vanish on the boundary of a square. Finally, one must transform the angular part of the Laplacian into the new variables which yields
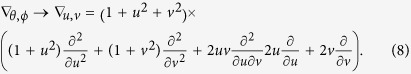


By the procedure outlined above we have transformed the problem of solving a harmonic oscillator problem in a non-trivial geometry, i.e. the red region in Fig. 2b), into solving a very simple boundary value problem
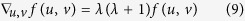
with 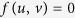 for 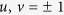. We write the eigenvalue in this way so it matches the usual 3D angular eigenvalue 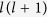. The equation for 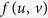 may be straighforwardly solved by using a two-dimensional Fourier expansion of the wave function. This will produce some spurious solutions as we must also impose bosonic symmetry among the two 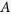 and two 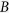 particles separately. This translates to the requirement that the solution be symmetric when reflected across the two diagonals. Notice that for each eigenvalue of this problem, 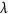, we obtain a whole class of solutions with energies 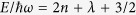 as we may add radial excitations.

The low-lying solutions of Eq. (9) are given in Table. 1. State number 1, 4, 6, 11, 13, and 15 have the required bosonic symmetries for the balanced system. A number of doubly degenerate states in the spectrum may be used to construct eigenfunctions for a four-body system with 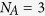 and 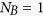 (or vice versa). In this case the wave function must vanish on the diagonal of the 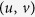 square domain which is achievable by taking proper linear combinations. The states marked ‘fermions’ in Table. 1 are antisymmetric across the two diagonals in the 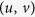 square and provide allowed states for all four-body two-component Fermi systems, i.e. 2+2, 3+1 or four identical fermions. The eigenenergies of the fermionic states have the exact values 7.5, 10.5 and 11.5. Our results differ by 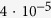 which attests to the accuracy of our method. All states in Table. 1 have been obtained using a modest 400 Fourier basis states. Notice that even though the lowest perfectly antiferromagnetic state for 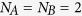 bosons is at the same energy as the fermionic state number 5 in Table. 1, they are not related since states with the configurations 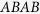 or 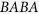 solve a different boundary value problem (corresponding to the yellow regions in Fig. 2b).

The energies obtained using this (semi)-analytical approach for the 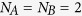 system are given in Fig. 2a) as triangles at 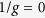. The two lowest triangles correspond to the angular ground state (lowest 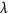 value) with 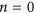 and 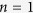. The two upper triangles are the first and second excited angular solutions both with 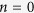. All four solutions have the spatial structure 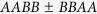. The blue dots in Fig. 4a) show the ground state density obtained by the transformation method discussed here. The rest of the spectrum at 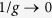 can be obtained by solving the boundary value problem in the green (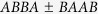) and yellow areas (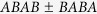). In the latter case a fermionized (totally antisymmetric) wave function is a solution. Our main interest here is to understand the ground state so we leave the remaining states and regions for future investigations.

### Analytics for imbalanced systems

The analytics provided here is applied for the Bose polaron, 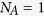 and 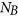 arbitrary, but can be extended to other systems. The Hamiltonian can be written
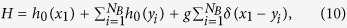
where 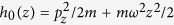 is a 1D harmonic oscillator. The 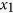 coordinate denotes the single 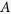 particle, the ‘impurity’, while 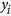 denotes the coordinates of the majority 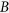 particles. We introduce an adiabatic decomposition of the total wave function of the form
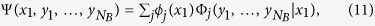
where 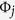 is a normalized eigenstate of the eigenproblem 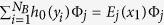 which depends parametrically on 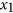. This expansion can be related to the Born-Oppenheimer approximation in which case one may consider 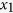 the ‘slow’ variable (typically the nuclear coordinate in molecular physics). In the limit of interest 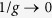, we impose the condition that the total wave function vanishes for 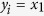, 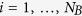. This implies that 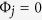 whenever 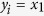. Since there are no intra-species interactions among the 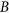 particles, we can write
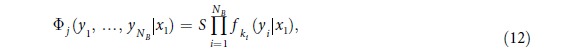
where 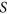 denotes the symmetrization operator and 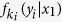 is the 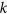
th normalized eigenstate of 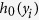 which satisfies the condition 
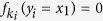
. The index 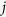 on 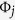 denotes the many different ways to distribute the 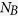 particles among the eigenstates of 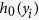 with the appropriate boundary condition. The Schrödinger equation for 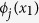 can now be written
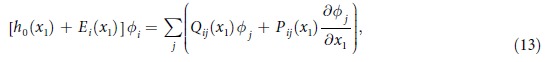
where.
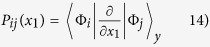

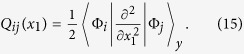


The subscript 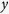 on the brackets denote integration over all 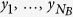. Note that 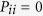 and 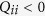
Ref. 47.

As we are interested in the ground state, we assume that all the 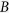 particles are in the same state, 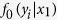, that we specify below. Since the nearest excited states are obtained by promoting one of the 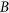 particles into a single-particle excited orbital, one can show that for large 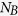 we can neglect all but the 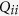 terms^48^. Furthermore, in the ground state we expect to find all the 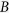 particles on one side of the impurity. If we assume that all 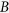 particles are to the left of the impurity, we can write the single-particle wave function, 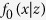 for 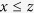, as
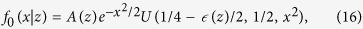
for 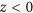 or 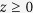 and 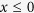, while for 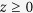 and 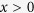 we write
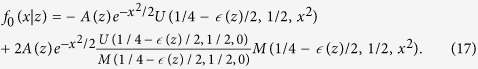
Here 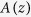 is a normalization factor, 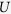 and 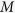 are the Tricomi and Kummer confluent hypergeometric functions, and we have used 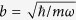 as the unit of length. Here 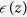 is a function that is chosen to satisfy the requirement 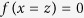. This is equivalent to finding the ground state solution of 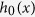 for 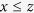 with the condition that the wave function must vanish at 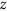.

Once we have determined the functions 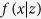 and 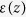, we can compute the adiabatic potential for the ground state. We have
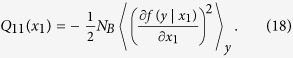


Furthermore, 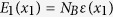 by additivity. The Schrödinger equation for 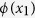 is then
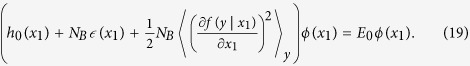


Note that the energy 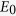 provides a variational upper bound to the exact energy.

The energies computed via this method for the polaron are shown in Fig. 6 and agree with the numerical results to within a few percent for the largest particle numbers in the figure. We expect the agreement to become better for even larger particle numbers. Furthermore, since we obtain the full wave function in an analytical form we may also compute the densities of both impurity and majority components. In Fig. 5a) we show the impurity densities for 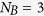 and 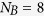, while Fig. 5d) shows the corresponding majority density. We see a striking agreement between the numerical results and the analytically tractable model presented here. The model presented here can be extended to excited states and also to systems with 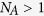.

### Tunneling experiments

For 1D few-body systems it is now possible to experimentally access the ratio of tunnelling probabilities for particles with different spins^16^. For strongly interacting fermionic systems this ratio can be obtained in a rather simple way without knowing the parameters of the experiment (e.g. height of the barrier), as it is determined solely by the probability for the particle to be at the edge of the trap^43^. For strongly interacting bosonic systems this is unfortunately no longer the case and the detailed parameters of the experiment are needed. To illustrate this consider a system with one impurity in a sea of 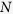 majority bosons. For 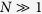 we have shown that for the ground state the impurity is pushed to the edge of the trap whereas the 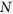 bosons can be accurately described using only the lowest energy level of the harmonic trap. Let us assume that the trap is lowered on one side. If the impurity sits on this side we will detect an impurity after some time 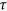 which may be very short, since the impurity is very close to the barrier. If, on the contrary, the impurity sits on the other side of the trap we will detect a majority particle after time 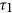. This time may be exponentially enhanced since tunnelling is from the ground state of the trap and the barrier is consequently large. The only system where the ratio of probabilities does not depend on the geometry of the experiment is the 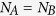 system where symmetry dictates that one will detect 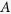 particles as frequently as 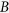 particles. As the perfectly antiferromagnetic state is only present for 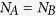 or 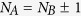, its tunneling signature is similar to many other states in the spectrum and detection of the state via tunneling would be quite difficult. For the excited states the ratio of probabilities is again very dependent on the parameters of the experiment. For example for the impurity and 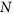 bosons system if the experiment is constructed such that only one particle can tunnel then there generally will be many states where only majority particles will be detected. This happens for the states where the impurity sits closer to the center of the trap.

## Additional Information

**How to cite this article**: Dehkharghani, A. *et al.* Quantum magnetism in strongly interacting one-dimensional spinor Bose systems. *Sci. Rep.* **5**, 10675; doi: 10.1038/srep10675 (2015).

## Figures and Tables

**Table 1:** Low-lying spectrum of Eq. (9). The last column denotes the specific system for which the particular solution is an allowed eigenstate using the notation 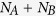 for bosons. See the text for details.

**State**	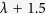	**System**
1	3.88989	2+2 bosons
2	5.64323	3+1 bosons
3	5.64323	3+1 bosons
4	7.07740	2+2 bosons
5	7.50002	fermions
6	7.60232	2+2 bosons
7	8.76318	3+1 bosons
8	8.76318	3+1 bosons
9	9.52342	3+1 bosons
10	9.52342	3+1 bosons
11	10.21574	2+2 bosons
12	10.50004	fermions
13	10.73383	2+2 bosons
14	11.50004	fermions
15	11.51706	2+2 bosons
16	11.85409	3+1 bosons
17	11.85409	3+1 bosons

## References

[b1] Bethe H. A. Zur Theorie der Metalle. I. Eigenwerte und Eigenfunktionen der linearen Atomkette. Z. Physik 71, 205–226 (1931).

[b2] Lieb E. H. & Liniger W. W. Exact analysis of an interacting Bose gas. The general solution and the ground state. Phys. Rev. 130, 1605–1616 (1963).

[b3] McGuire J. B. Interacting Fermions in One Dimension. I. Repulsive Potential. J. Math. Phys. 6, 432–439 (1965).

[b4] McGuire J. B. Interacting Fermions in One Dimension. II. Attractive Potential. 7, 123–132 (1966).

[b5] Yang C. N. Some Exact Results for the Many-Body Problem in one Dimension with Repulsive Delta-Function Interaction. Phys. Rev. Lett. 19, 1312–1315 (1967).

[b6] Lieb E. H. & Wu F. Y. Absence of Mott Transition in an Exact Solution of the Short-Range, One-Band Model in One Dimension. Phys. Rev. Lett. 20, 1445–1448 (1968).

[b7] Haldane F. D. M. Effective Harmonic-Fluid Approach to Low-Energy Properties of One-Dimensional Quantum Fluids. Phys. Rev. Lett. 47, 1840 (1981).

[b8] Haldane F. D. M. ‘Luttinger liquid theory’ of one-dimensional quantum fluids. I. Properties of the Luttinger model and their extension to the general 1D interacting spinless Fermi gas. J. Phys. C: Solid State Phys. 14, 2585 (1981).

[b9] Giamarchi T. Quantum Physics in One Dimension (Oxford University Press Inc., New York, 2003).

[b10] Cazalilla M. A., Citro R., Giamarchi T., Orignac E. & Rigol M. One dimensional bosons: From condensed matter systems to ultracold gases. Rev. Mod. Phys. 83, 1405 (2011).

[b11] Paredes B. *et al.* Tonks-Girardeau gas of ultracold atoms in an optical lattice. Nature 429, 277–281 (2004). 15152247 10.1038/nature02530

[b12] Kinoshita T., Wenger T. & Weiss D. S. Observation of a One-Dimensional Tonks-Girardeau Gas. Science 305, 1125–1128 (2004). 15284454 10.1126/science.1100700

[b13] Kinoshita T., Wenger T. & Weiss D. S. A quantum Newton’s cradle. Nature 440, 900–903 (2006). 16612376 10.1038/nature04693

[b14] Haller E. *et al.* Realization of an Excited, Strongly Correlated Quantum Gas Phase. Science 325, 1224–1227 (2009). 19729651 10.1126/science.1175850

[b15] Serwane F. *et al.* Deterministic Preparation of a Tunable Few-Fermion System. Science 332, 336–338 (2011). 21493855 10.1126/science.1201351

[b16] Zürn G. *et al.* Fermionization of Two Distinguishable Fermions. Phys. Rev. Lett. 108, 075303 (2012). 22401221 10.1103/PhysRevLett.108.075303

[b17] Wenz A. N. *et al.* From Few to Many: Observing the Formation of a Fermi Sea One Atom at a Time. Science 342, 457 (2013). 24159041 10.1126/science.1240516

[b18] Pagano G. *et al.* A one-dimensional liquid of fermions with tunable spin. Nature Phys. 10, 198–201 (2014).

[b19] Recati A., Fedichev P. O., Zwerger W. & Zoller P. Spin-Charge Separation in Ultracold Quantum Gases. Phys. Rev. Lett. 90, 020401 (2003). 12570530 10.1103/PhysRevLett.90.020401

[b20] Kuklov A. B. & Svistunov B. V. Counterflow Superfluidity of Two-Species Ultracold Atoms in a Commensurate Optical Lattice. Phys. Rev. Lett. 90, 100401 (2003). 12688982 10.1103/PhysRevLett.90.100401

[b21] Duan L.-M., Demler E. & Lukin M. D. Controlling Spin Exchange Interactions of Ultracold Atoms in Optical Lattices. Phys. Rev. Lett. 91, 090402 (2003). 14525163 10.1103/PhysRevLett.91.090402

[b22] Eisenberg E. & Lieb E. H. Polarization of Interacting Bosons with Spin. Phys. Rev. Lett. 89, 220403 (2002). 12485054 10.1103/PhysRevLett.89.220403

[b23] Nachtergaele B. & Shannon S. Ferromagnetic Lieb-Mattis Theorem. Phys. Rev. Lett. 94, 057206 (2005). 15783691 10.1103/PhysRevLett.94.057206

[b24] Zöllner S., Meyer H.-D. & Schmelcher P. Composite fermionization of one-dimensional Bose-Bose mixtures. Phys. Rev. A 78, 013629 (2008).

[b25] Hao Y. & Chen S. Density-functional theory of two-component Bose gases in one-dimensional harmonic traps. Phys. Rev. A 80, 043608 (2009).

[b26] Garcia-March M. A. & Busch Th. Quantum gas mixtures in different correlation regimes. Phys. Rev. A 87, 063633 (2013).

[b27] Garcia-March M. A. *et al.* Sharp crossover from composite fermionization to phase separation in microscopic mixtures of ultracold bosons. Phys. Rev. A 88, 063604 (2013).

[b28] Garcia-March M. A. *et al.* Quantum correlations and spatial localization in one-dimensional ultracold bosonic mixtures. New J. Phys. 16, 103004 (2014).

[b29] Zinner N. T. *et al.* Fractional energy states of strongly-interacting bosons in one dimension. Europhys. Lett. 107, 60003 (2014).

[b30] Sutherland B. Further Results for the Many-Body Problem in One Dimension. Phys. Rev. Lett. 20, 98 (1968).

[b31] Li Y.-Q., Gu S.-J., Ying Z.-J. & Eckern U. Exact results of the ground state and excitation properties of a two-component interacting Bose system. Europhys. Lett. 61, 368 (2003).

[b32] Fuchs J. N., Gangardt D. M., Keilmann T. & Shlyapnikov G. V. Spin Waves in a One-dimensional Spinor Bose Gas. Phys. Rev. Lett. 95, 150402 (2005). 16241701 10.1103/PhysRevLett.95.150402

[b33] Guan X., Batchelor M. T. & Takahashi M. Ferromagnetic behavior in the strongly interacting two-component Bose gas. Phys. Rev. A 76, 043617 (2007).

[b34] Halperin B. I. & Hohenberg P. C. Hydrodynamic Theory of Spin Waves. Phys. Rev. 188, 898 (1969).

[b35] Halperin B. I. Dynamic properties of the multicomponent Bose fluid. Phys. Rev. B 11, 178 (1975).

[b36] Zvonarev M. B., Cheianov V. V. & Giamarchi T. Spin Dynamics in a One-Dimensional Ferromagnetic Bose Gas. Phys. Rev. Lett. 99, 240404 (2007). 18233427 10.1103/PhysRevLett.99.240404

[b37] Akhanjee S. & Tserkovnyak Y. Spin-charge separation in a strongly correlated spin-polarized chain. Phys. Rev. B 76, 140408 (2007).

[b38] Matveev A. & Furusaki A. Spectral Functions of Strongly Interacting Isospin-1/2 Bosons in One Dimension. Phys. Rev. Lett. 101, 170403 (2008). 18999725 10.1103/PhysRevLett.101.170403

[b39] Kamenev A. & Glazman L. Dynamics of a one-dimensional spinor Bose liquid: A phenomenological approach. Phys. Rev. A 80, 011603(R) (2009).

[b40] Caux J., Klauser A. & van den Brink J. Polarization suppression and nonmonotonic local two-body correlations in the two-component Bose gas in one dimension. Phys. Rev. A 80, 061605 (2009).

[b41] Lindgren E. J., Rotureau J., Forssén C., Volosniev A. G. & Zinner N. T. Fermionization of two-component few-fermion systems in a one-dimensional harmonic trap. New J. Phys. 16, 063003 (2014).

[b42] Rotureau J. Interaction for the trapped fermi gas from a unitary transformation of the exact two-body spectrum. Eur. Phys. J. D 67, 153 (2013).

[b43] Volosniev A. G., Fedorov D. V., Jensen A. S., Valiente M. & Zinner N. T. Exact solution of strongly interacting confined quantum systems in one dimension. Nature Communications 5, 5300 (2014). 10.1038/ncomms6300 25366925

[b44] Girardeau M. D. Relationship between systems of impenetrable bosons and fermions in one dimension. J. Math. Phys. 1, 516–523 (1960).

[b45] Girardeau M. D. & Olshanii M. Theory of spinor Fermi and Bose gases in tight atom waveguides. Phys. Rev. A 70, 023608 (2004).

[b46] Busch T., Englert B.-G., Rzażewski K. & Wilkens M. Two cold atoms in a harmonic trap. Found. Phys. 28, 549–559 (1998).

[b47] Nielsen E., Fedorov D. V., Jensen A. S. & Garrido E. The three-body problem with short-range interactions. Phys. Rep. 347, 373–459 (2001).

[b48] Dehkharghani A. S., Volosniev A. G. & Zinner N. T. Quantum impurity in a one-dimensional trapped Bose gas. *Preprint* arXiv:1503.03725 (2014).

